# Case Report: Endoscopic evacuation of acute subdural hematoma in selected young patients: a report of two cases

**DOI:** 10.3389/fsurg.2026.1861967

**Published:** 2026-07-24

**Authors:** Hao Guo, Heng Guo, Hong Zhou, Yue Gong, Hui Jiang, Di Wu, Qiang Xiang

**Affiliations:** 1 Department of Emergency Medicine, The First Affiliated Hospital, Army Medical University, Chongqing, China; 2 Department of Neurosurgery, General Hospital of Western Theater Command, Chengdu, Sichuan, China

**Keywords:** acute subdural hematoma, endoscope, hemostasis, working space, young patients

## Abstract

**Background:**

Endoscope-assisted surgery for acute subdural hematoma (ASDH) has been successfully performed in selected elderly patients. Compared with a large craniotomy or craniectomy, this technique is characterized by minimal invasiveness, mild operative hemorrhage, and short duration. However, endoscopy does not seem to be appropriate for ASDH in young patients without brain atrophy, resulting in a scarcity of reports for this cohort.

**Case presentation:**

We present two young cases in which both ASDHs were effectively and safely evacuated endoscopically. The two men were 39 and 31 years old. Both patients maintained a stable neurological status before surgery but met the surgical indications.

**Conclusion:**

This favorable outcome suggests that endoscopic evacuation of ASDH could be proposed as an alternative option for selected young patients with clinical and radiological stability.

## Introduction

Acute subdural hematoma (ASDH) is the most prevalent pathology associated with traumatic brain injury (TBI) (1). ASDHs causing a remarkable mass effect typically warrant immediate surgical evacuation. Early removal of space-occupying lesions can reduce intracranial hypertension and prevent brain herniation. The mainstay of surgical treatment is hematoma removal through a large craniotomy or decompressive craniectomy (2). However, it remains unclear whether either is associated with better outcomes for ASDH. When craniotomy is chosen as a management option in appropriately selected ASDH cases, a less invasive alternative may be a good choice. Neuroendoscopy through a small bone flap can accommodate minimally invasive instruments and achieve the safe removal of hematoma, as evidenced by favorable results in treating elderly patients with ASDH (3). The shrinking volume within the elderly cranial vault provides technical feasibility for a series of endoscopic performances. Nevertheless, in younger patients without brain atrophy, it is worth studying whether endoscopic surgery for ASDH could still demonstrate technical superiority. Similar reports are rare (4, 5). Herein, we present the endoscopic procedure for ASDH in two young patients without preoperative deterioration and discuss the treatment challenges.

## Case presentation

### Case 1

**Figure 1:**
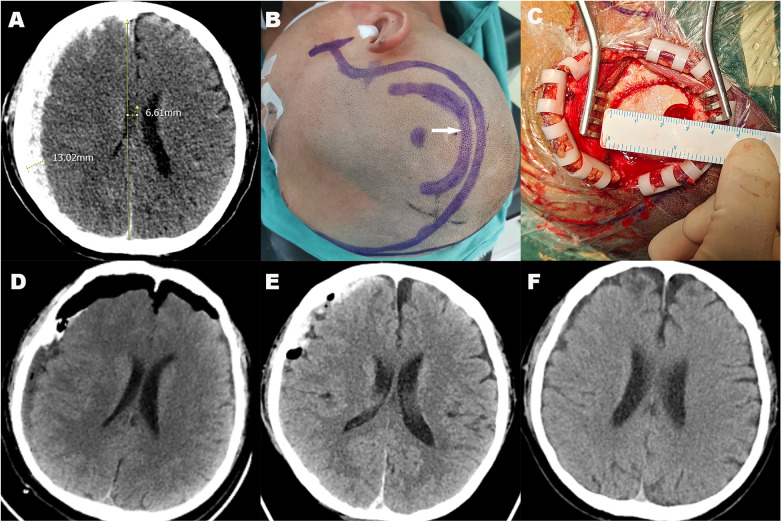
**(A)** Head CT scan showing a thick acute right-sided subdural hematoma and remarkable midline shift at the time of admission. **(B)** Curvilinear incision (white arrow) could facilitate conversion to a possible craniotomy. **(C)** The measured size of the bone flap was 3 cm. **(D–F)** Follow-up CT on immediate postoperation, 4, and 25 days later, respectively.

A 39-year-old man with a history of schizophrenia and chronic alcoholism presented with a severe headache for 11 h and an epileptiform seizure after falling at home. A brain computed tomography (CT) scan showed an acute subdural hematoma with a thickness of 13.02 mm in the right frontotemporoparietal region, a midline shift of 6.61 mm, compression of the right ventricle, and traumatic subarachnoid hemorrhage (SAH) (Figure 1A). The patient's vital signs were stable, and the Glasgow Coma Scale (GCS) was 15. After being admitted to the trauma intensive care unit, he gradually developed delirium, agitation, and aggression. Although the patient's abnormal behavior might be highly related to his history, we had to first attribute it to the elevated intracranial pressure. In the absence of herniation, endoscopic surgery with general anesthesia was scheduled 40 h later with no progressive findings on repeated CT before surgery. The patient was placed in the lateral position. A curvilinear scalp incision was made to locate the thickest portion of the hematoma, partly coinciding with the conventional reverse question mark approach, which could facilitate conversion to craniotomy (Figure 1B). A 3-cm-diameter bone flap was created (Figure 1C). The dura was incised in a cruciform after being suspended, exposing a dark hematoma around the opening, which was removed under direct vision and irrigated with saline. Then, a rigid endoscope with a 0° angle and a 4 mm diameter (Karl Storz Endoskope, Tuttlingen, Germany) was introduced. Initially, the space of the subdural collection was too narrow to maneuver the endoscope and irrigation suction cannula deeper until air was gradually displaced into the hematoma cavity in the lateral position with no tilt. Almost all hematomas were evacuated. After saline irrigation and endoscopy revealed no other bleeding points, a silicone drain was inserted into the subdural space. The dura mater was closed, the bone flap was repositioned, and the skin was closed. Postoperative CT showed adequate hematoma resolution and a significant decrease in the mass effect (Figures 1D–F). The patient's headache notably alleviated, and the aberrant manifestations disappeared immediately. After an additional 9 days of postoperative care, the patient was uneventfully discharged.

### Case 2

**Figure 2:**
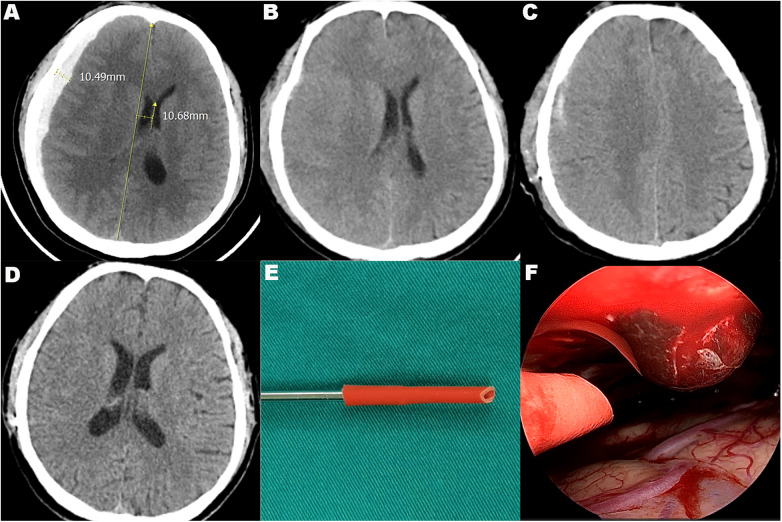
**(A)** Head CT scan at admission depicting an acute subdural hematoma and pronounced midline shift. **(B,C)** CT on postoperative day 4 showed sufficient hematoma removal and residual hematoma caused by venous bleeding intraoperatively. **(D)** The midline shift improved 46 days after surgery. **(E,F)** The rubber tip connected to the suction cannula was clipped into a slope shape to prevent brain damage.

A 31-year-old man with a prolonged history of alcohol consumption experienced a headache for 6 months. He took one or two packs of analgesic compounds (including 230 mg aspirin per pack) for relief every two or three days. Two months ago, he accidentally bumped his head against a solid object while welding. A similar scenario occurred 3 days before. On admission, the patient complained of a headache, with the analgesic compound being suspended for 3 days. His GCS score was 15. No other neurological signs were observed. The initial head CT scan showed a thick mass (10.49 mm) of subdural high intensity located over the right hemisphere, with a midline shift (10.68 mm) and SAH (Figure 2A). Surgical evacuation was indicated, and endoscopic surgery was delayed for another 4 days for the functional recovery of platelets, during which the headache continued in a relatively stable process. The presence of two bumps in the past two months, irregular administration of antiplatelet drugs, and an unchanged level of consciousness complicated the evaluation of when the hematoma occurred. Intraoperatively, a typical outer membrane structure of a chronic subdural hematoma (CSDH) under the dura mater was not detected, other than the clotted hematoma, which confirmed that this case was actually an acute subdural hematoma. On the third day after the operation, the patient reported no headache. Postoperative CT showed sufficient hematoma removal (Figures 2B–D).

## Discussion

Herein, we add another report on endoscopic evacuation for ASDH in young patients to the sparse literature. Our results revealed that neuroendoscopy through a small bone flap could accommodate minimally invasive instruments and achieve safe removal of ASDH in selected young patients. For high-intensity subdural hematomas without an exact time of injury, endoscopy can facilitate the differential diagnosis of acute or chronic SDH. The two cases presented some unique features that we considered during endoscopy.

The indications for endoscopic surgery for ASDH have not yet been established due to the small size of relevant studies and heterogeneity between them (3). Among all the factors to consider for eligibility criteria, the key variables are age, radiological features, and baseline neurological status. For elderly patients in the cohort, symptomatic and isolated ASDHs in the absence of enlarging hematomas or large parenchymal lesions are optimal candidates (6). The nature of the less compensable space in the cranial cavity of young adults may necessitate a more restricted patient selection. Cases 1 and 2 primarily showed ASDH and SAH, apart from a localized contusion in Case 1. Both reached the surgical indication that an ASDH thicker than 10 mm or a midline shift beyond 5 mm is required according to the guideline (7). Both patients maintained a stable neurological status, which allowed for watchful waiting prior to endoscopic surgery. Brain herniation, which may warrant an emergent craniotomy or craniectomy, did not occur in either case. These shared characteristics may be the most significant determinants for the feasibility of endoscopy. Kuge et al. first applied endoscopy unintentionally but successfully to a 31-year-old patient with a massive ASDH (5). The patient presented with a GCS of 6 and anisocoria, indicating brain herniation. After osmotherapy and emergency trepanation, anisocoria disappeared. Nevertheless, an operating theater was not available for a craniotomy at the same time, so an endoscopic procedure was chosen to be performed in an emergency room under local anesthesia. Their treatment was effective and achieved a good outcome, but it may pose a high risk to the patient, especially when a standard craniotomy is strongly indicated. Instead, it could be inferred from our study that endoscopic surgery may be more applicable to young patients presenting with a high and stable preoperative GCS, as supported by evidence from studies on the elderly population (8, 9).

The key to endoscopic procedures is to ensure an adequate working space. ASDH, indicative of surgery, typically covers the contour of the hemisphere in a crescent shape. The cramped nature of the subdural space obviously constrains the establishment of a working passage, which is particularly more evident in young patients with normal brain volume or under rapid brain reexpansion after the removal of ASDH. The present cases indicate two methods for initiating and enlarging the working space. The first case utilized proper positioning, which allowed more air to enter and fill the space easily. In the second case, we primarily used mannitol to reduce brain volume. Regardless of the method used, our study proposed the necessity and importance of the institution of artificial pneumocephalus for acute subdural clot removal. Air accumulation in the postoperative subdural cavity has been observed in most cases in the majority of related studies. The expandable space created by artificial pneumocephalus is not comparable to the artificial pneumoperitoneum used in laparoscopic surgery, but it has indeed proven to be very useful. Although a thicker hematoma may indicate a wider surgical space under the same conditions, the actual working space would be influenced immediately by reexpansion of the compressed brain after clot removal.

The size of the bone flap is another important factor closely related to the working space. A larger diameter appears more invasive, while a smaller diameter indicates an insufficient working path for the rigid endoscope. Identifying an absolute size threshold may not be as significant due to various hematoma configurations or heterogeneous bony structures. Most reports recommend a diameter between 2 and 5 cm for rigid endoscopy in elderly patients with ASDH (3, 10, 11). There are two methods for optimizing the size. One is to initially choose a slightly larger flap when conducting this technique, then gradually explore a minimal craniotomy through cases. The other is to bevel along the outer table and/or the inner table using abrasive drilling to allow for a maximal working angle of the endoscope (12). For a flexible endoscope, the bone window might be smaller because the scope can provide a broader vision when turning the tip, although a growing number of rigid endoscopes have been applied in ASDH.

The limited operating space under the endoscope poses a huge challenge to fresh bleeding control. In contrast, hemostasis under direct visual inspection is readily available in traditional craniotomy. The origin of bleeding in most ASDHs results from venous vascular injury adjacent to the brain surface (13). When the hematoma enlarges to some extent, the regional pressure adjacent to the venous injury is elevated and maintained, which may further occlude the break or laceration and stop the hemorrhage. Therefore, we sometimes cannot find the bleeding point after removing the acute subdural hematoma entirely in conventional craniotomies. Robinson et al. reported factors associated with early ASDH expansion (<72 h from injury) (14), but the public literature has not clarified when ASDHs can no longer expand, which means achieving potential bleeding control. Here, we considered ASDHs without further expansion on CT scans at a certain interval as stable, whereas ever-expanding hematomas were considered unstable (9). Whether the hematoma is stable is also consistent with the neurological status. For the hematoma evacuation procedure, stable ASDHs might require fewer substantial hemostatic maneuvers apart from clot suction. Unstable ASDHs probably require extra direct performance toward bleeding points, either hemostasis by compressing the venous bleeding or bipolar cautery, which may be more difficult to conduct in a narrow passage under endoscopy. Consequently, the priority is to identify whether the hematoma is stable and choose the corresponding management. If surgery is performed in advance, it can cause fresh bleeding and disturb the vision of the endoscope. However, a delayed procedure may lead to a persistent mass effect and increasingly serious intracranial hypertension, which would mandate traditional craniotomy. The stability of the hematoma may determine the choice of surgical strategy and the degree of operative difficulty under endoscopy. Ideally, ASDH could be evacuated by endoscopic suction only, without the need for additional procedures, as demonstrated in Case 1. In Case 2, we used gelatin packing for hemostasis when venous bleeding occurred, which resulted in a residual hematoma and prolonged length of stay.

With respect to the surgical approach, we chose a partial route of frontotemporoparietal standard craniotomy near the thickest hematoma, often in the parietal zone. This is because if hemostasis could not be achieved endoscopically or if bone decompression was required, the skin incision could be conveniently extended or bridged. To reduce the risk of hemorrhage, several techniques have been developed. Tanaka et al. used protective sheets to cover the brain surface to avoid iatrogenic injury (11). Other researchers have applied malleable suction to retract the brain, with its tip pointing to the dura (5). In this procedure, we clipped the rubber tip connected to the suction cannula into a slope shape, which can safely and effectively aspirate the hematoma away with the slope in the direction of the dura instead of the brain surface (Figures 2E,F).

## Conclusion

The present cases suggest that endoscopic evacuation of ASDH could be prudently applied to selected young patients with a stable preoperative status, where surgically indicated. Strict inclusion criteria, institution of a sufficient working path, and effective hemostasis management under endoscopy could achieve favorable outcomes. Neuroendoscopy as a treatment option may require continuous refinement and warrants further investigation in selected young patients with ASDH.

## Data Availability

The original contributions presented in this study are included in the article/Supplementary Material. Further inquiries can be directed to the corresponding authors.
